# A High Reliability 3D Scanning Measurement of the Complex Shape Rail Surface of the Electromagnetic Launcher

**DOI:** 10.3390/s20051485

**Published:** 2020-03-08

**Authors:** Zhaoxin Wang, Baoming Li

**Affiliations:** National Key Laboratory of Transient Physics, Nanjing University of Science and Technology, Nanjing 210094, China; JessieWang1105@outlook.com

**Keywords:** 3D scanning, binocular stereovision, laser dot projection, rail surface, railgun

## Abstract

After an electromagnetic railgun launch, a series of damage phenomena may cause the inner bore surface to become complex, such as gouging and deposition. Furthermore, the rail surface will be uneven and blackened by oxidation. To understand these forms of rail degradation, many previous studies have mentioned several surface scanning methods, but none of these can be used in the complex inner bore. Therefore, we present a 3D scanning system based on binocular stereovision technology combined with the active illumination, which can be used to obtain the rail surface topography under a complex inner bore environment. The laser dot projection is applied as the active illumination. In contrast with other active illumination, laser dot projection has high reconstruction reliability. By combining laser dot projection with binocular stereovision, the object can be completely reconstructed. In addition, an image acquisition method which can improve image signal-to-noise ratio is proposed. The proof-of-principle experiment of the system is done under dim light conditions. Through the experiment, the 3D depth map of the rail surface is obtained and the gouge crater is scanned out. Meanwhile, system evaluation and measurement uncertainty analysis have also been carried out.

## 1. Introduction

**Figure 1:**
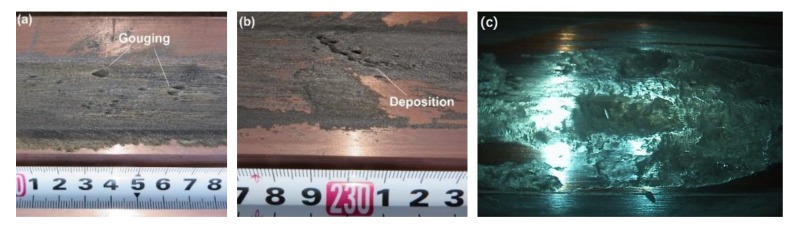
The appearance of the rail’s surface. (**a**) Gouging. (**b**) The deposition on the rail surface. (**c**) The complex inner bore surface.

With the development of the railgun, the interior ballistic performance and rails’ lifetime have attracted increasing attention. A railgun is a type of electromagnetic launcher (EML). It converts electromagnetic energy into kinetic energy, greatly guiding the armature and moving the projectile [1]. During the process of a railgun launch, the launch package is accelerated down the length of the rails. For a larger launch package mass and a higher launch package velocity, there will be a large current across the interface between the rails and the armature. As the armature moves in the bore, the rails experience high electrical current densities, large electromagnetic loads, and high sliding velocities, which can cause friction and electrical heating. These conditions may cause the inner bore surface to become complex; some are shown in Figure 1, including gouging (see Figure 1a) [2] and deposition (see Figure 1b) [3,4]. In addition, these forms of rail degradation have been detrimental to the rails [5], which can not only shorten the rails’ lifetime but also affect the interior ballistic performance. Therefore, it is seriously important to understand these different forms of rail degradation.

Over the past few decades, many researchers have devoted themselves to better understanding the surface appearance of the rails. In addition, their previous studies have mentioned several surface profile measurement technologies. A WYKO rough surface tester, which was a non-contact optical profiler that blended vertical scanning interference microscopy and digital signal processing, was utilized by Persad et al. as early as 1997 [2,3], to produce the selected surface contour at the specified position along the wavy deposit [3] and a three-dimensional view of the gouge [2]. In 2005, Meger et al. used a KLA-Tencor P-15 profilometer to measure the two-dimensional profile of the deposits on the rail surface [4]. Watt et al. employed a stylus profilometer to measure erosion damage [6] and the dimensions of the grooves [7]. Later, a 3-D Digital Optix 400s laser scanner was used to scan the rail surfaces after etching by Zielinski et al. [8]. These measurement technologies are all effective methods and can be better applied in individual rail surface measurement. However, they cannot be used in the complex inner bore in-between launches.

**Figure 2:**
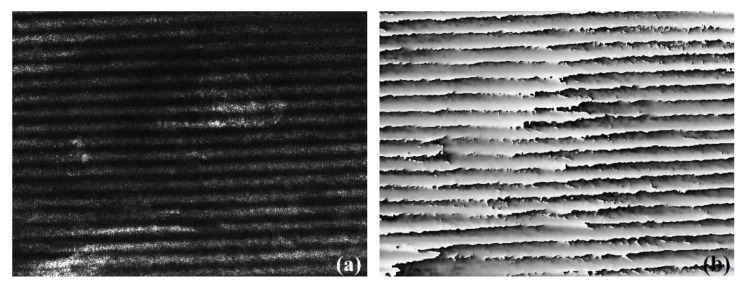
Sinusoidal fringe pattern projected onto the rail surface. (**a**) Sinusoidal fringe pattern projection on the rail surface. (**b**) The phase unwrapping result of figure (**a**).

The inner bore of the barrel is small in space and exists dim light inside. In addition, it has a complex inner bore surface, which is shown in Figure 1c. The friction and electrical heating make the rail surface concave-convex and oxidized. Due to the effect of the complex inner bore environment, many 3D profile measurement methods cannot be used effectively. Considering the dim light condition, the first thought is active illumination, such as binary-coded pattern [9,10] and sinusoidal fringe pattern [11,12,13,14]. The binary coding technique uses black-and-white stripes to form projection patterns. However, it is less sensitive to the surface characteristics, because only binary values exist in pixels [15]. However, the sizes of the rail surface characteristics of a railgun are usually millimeter in scale or even micrometer in scale, either of which requires a measuring method with high sensitivity. That is, the binary coding technique is not applicable to the rail surface. When the sinusoidal fringe pattern is used as active illumination, the 3D reconstruction method can be phase shifting [11,12] or Fourier transform profilometry (FTP) [13,14]. They both have satisfactory sensitivity. However, they have high requirements for the accuracy of the fringe pattern shown in the image. This is because no matter whether it is phase shift or FTP, the premise of obtaining accurate results is the accuracy of phase modulation. Additionally, in order to get more accurate phase modulation results, the fringe pattern must be more precise. Figure 2a shows the sinusoidal fringe pattern projected onto the rail surface. According to Figure 2a, we can find that the sinusoidal fringe pattern is not ideal because of the complex inner bore surface (such as the complex reflection on a metal surface). The fringe pattern has a dark area and a bright area, and the brightness of the whole pattern is not uniform. Moreover, the deformation and the edge of the sinusoidal fringe pattern are not obvious, which will affect the calculation results, and the existing FTP algorithm could not solve them well. Figure 2b shows the phase unwrapping result of Figure 2a, and we can see that the unwrapping result is not enough for full reconstruction, and the sinusoidal fringe pattern projection is not satisfactory. In addition, through many experiments, we found that the sinusoidal fringe pattern was seriously affected by overexposure during acquisition, which could not be easily controlled. Therefore, for the rail surface, we are not going to consider using these three methods.

As seen from Figure 1c and Figure 2a, the uneven surface which is also blackened by oxidation makes the reflection angles in the whole areas of fringe pattern projection inconsistent, which leads to the nonuniform brightness. That is, individual active illumination is more susceptible to the effect of the complex surface environment. Now that using active illumination only is not appropriate, we can try to combine several methods. Binocular stereo vision measurement [16] is a 3D scanning method that is not easily affected by the environment. It is also a non-contact method, and has the advantages of high efficiency, good accuracy, simple system structure, and good adaptability. However, due to the complex inner bore environment, there is a problem of binocular matching under weak light environment. It is necessary to think about how to overcome it. Combining active illumination is feasible, but we will exclude the fringe pattern projection. Therefore, to overcome the matching problem in dim light, the laser dot projection is used as active illumination. Here, we report on a combined scanning system based on the binocular stereovision theory and laser dot projection, applicable to 3D profile measurement of rail under dim light. The key role of the laser dot projection is matching. In order to get more accurate images which contain laser dot projection, an image acquisition method is proposed. Moreover, we measured a part of the surface of the rail containing a gouge crater after launching by using our proposed 3D scanning system. Additionally, we did an analysis for the accuracy of the distance between cameras and the rail surface.

## 2. Scanning System

**Figure 3:**
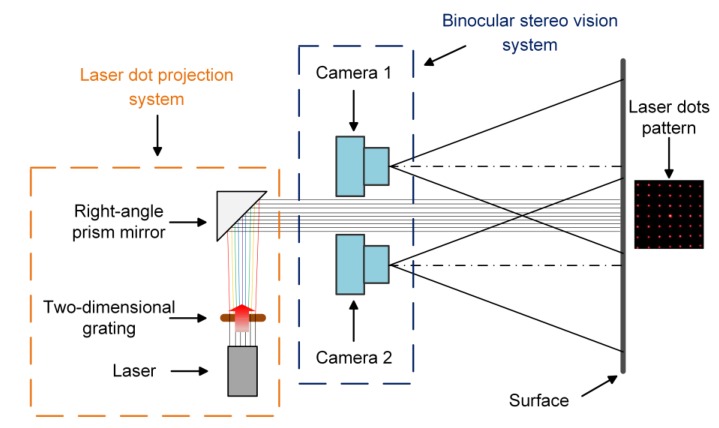
The schematic of the 3D scanning system.

The 3D scanning system is composed of two parts, a binocular stereo vision system and a laser dot projection system. Figure 3 shows the schematic of our 3D scanning system. The binocular stereo vision system consists of two cameras; and the laser dot projection system consists of a laser device, a right-angle prism mirror, and a two-dimensional grating. In Figure 3, the laser passes through the two-dimensional grating, forming laser dots pattern as initiative light. Then the laser beam is reflected from the right-angle prism mirror to the surface, and laser dots pattern is projected onto the rail surface. After that, two cameras respectively capture two surface images.

### 2.1. Binocular Stereo Vision System

Binocular stereo vision [16] is a technique used for obtaining the 3D geometric information of an object from two slightly different images. In the system, two identical cameras with parallel optical axis observe one same object, and two images are acquired simultaneously. Based on the visual disparity, 3D depth information can be calculated by trigonometry relations. The results are presented in the form of depth maps. We adopt a parallel optical axis binocular system, which is a simple system. Additionally, the geometry parameters of the system are known by a camera calibration procedure determining the measurement accuracy.

**Figure 4:**
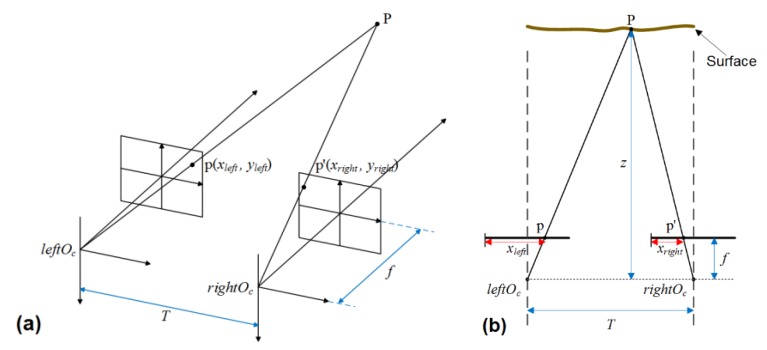
The parallel optical axis binocular system. (**a**) A simple schematic diagram of parallel optical axis binocular system. (**b**) The 2D plan of the schematic diagram.

The parallel optical axis binocular system is a more convenient configuration, in which corresponding points are constrained on the same image scanline. A simple schematic diagram of the parallel optical axis binocular system is shown in Figure 4a. To simplify the calculation, images are drawn in front of the optical center of the lens. The distance between image and the optical center of the lens is f, which stands for focal length. Moreover, the origin of the image’s coordinate system is located on the intersection of imaging plane and the optical axis. Suppose such P is the point of the surface to be measured, whose corresponding image points are $p(x_{left},y_{left})$ and $p\prime (x_{right},y_{right})$ respectively on the left and right image plane. $leftO_{c}$ and $rightO_{c}$ are the optical centers of the two cameras. T is the baseline, which represents the distance between two camera optical centers. z is the distance in viewing direction. For observations and calculations, the 2D plan of the schematic diagram is shown in Figure 4b.

With the trigonometry relations and by considering similar triangles in Figure 4b, the geometric relationship can be described as

(1) $$\frac{T-(x_{left}-x_{right})}{z-f}\;=\;\frac{T}{z}.$$

Then the distance can be expressed as

(2) $$z\;=\;\frac{fT}{x_{left}-x_{right}}\;=\;\frac{fT}{d},$$

where $d\;=\;x_{left}\;-\;x_{right}$ is the disparity between left and right images, which is the main basis to calculate the distance in binocular stereovision. According to Equation (2), we can see that the triangulation is a function of the disparity and the calibration parameters.

### 2.2. Laser Dot Projection System

**Figure 5:**
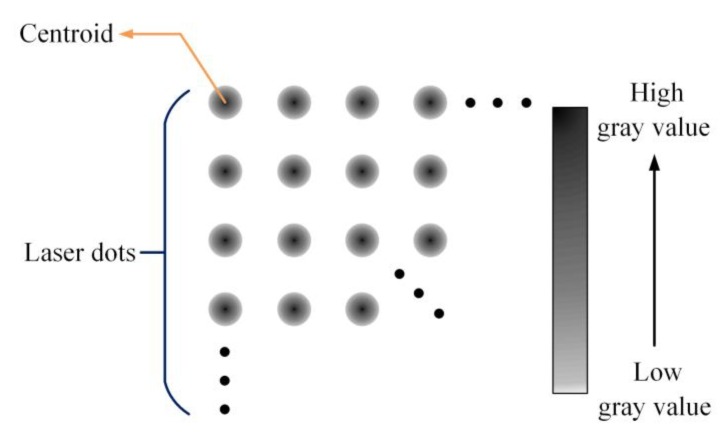
A filter is used to change the laser dots’ gray value, and the centroid pixels of the dots get the biggest value.

In computer vision, the use of active illumination makes 3D surface imaging more reliable and the acquired data easier to interpret [15]. In our work, the laser dot projection is used as active illumination, but its main purpose is to improve the efficiency of matching in dim light. When laser dots are projected on the surface, the dots’ positions are shifted in the image due to the surface variation and the camera position. From the dots’ positions and the geometry of the optical system, the surface contour can be deduced. In addition, as far as we know, laser beam exists a certain divergence angle, which makes the light intensity value in the center point of each dot be maximal. Therefore, the center point of each dot, which is the centroid of each dot, can be used for feature extraction and stereo matching. Considering that each dot actually contains multiple pixels, we think the centroid pixel is the feature point. Figure 5 shows the centroid pixels in the laser dots. In order to catch the centroids, we use a filter to change the dots’ gray value, and then the center point of the dot will get the biggest value. This is why we set the center point as the centroid.

The centroid algorithm is generally used in finding center points [17]. Here we assume a given image of size *M* × *N* pixels, where *M* and *N* are number of rows and columns in the image, respectively. The centroid coordinates are calculated as follows:

(3) $$x_{\mathrm{centroid}}=\frac{\sum_{j=1}^{N}\sum_{i=1}^{M}\left(I_{ij}\cdot x\right)}{\sum_{j=1}^{N}\sum_{i=1}^{M}I_{ij}},$$

(4) $$y_{\mathrm{centroid}}=\frac{\sum_{i=1}^{M}\sum_{j=1}^{N}\left(I_{ij}\cdot y\right)}{\sum_{j=1}^{N}\sum_{i=1}^{M}I_{ij}}$$

where $x_{centroid}$ and $y_{centroid}$ are the centroid coordinates in X direction and Y direction respectively; x, y denote the location of each pixel; and $I_{ij}$ is the column j and the row i’s pixel intensity.

## 3. High Signal-to-noise Ratio Image Acquisition

### 3.1. Image Acquisition Method

The signal-to-noise ratio (SNR) is one of the image quality evaluation methods. In 3D surface reconstruction, the SNR of the acquired image will affect the reconstruction accuracy. When the SNR value of the image is higher, the image quality will be better; then the accuracy of surface profile reconstruction can be improved. The SNR of the image can be written as

(5) $$SNR=10\log_{10}\left(\frac{\sigma_{f}^{2}}{\sigma_{n}^{2}}\right).$$

where $\sigma_{f}^{2}$ is the variance of the reference signal and $\sigma_{n}^{2}$ is the variance of the noise of the actual signal relative to the reference signal.

Due to the complex inner bore environment, the image signal obtained by scanning the rail surface is weak relative to background clutter and noise, which makes for a low image SNR. If the value of image signal is increased, the noise will be produced by the reflection of metal, because rails of the railgun are mostly made of metal alloys. Additionally, the reflection of metal will lead to the captured images being overexposed. Thus, an image acquisition method that can improve image SNR is illustrated here, which is based on the statistical characteristics of image noise and the removal of imaging background noise. Considering the statistical characteristics of image noise, while the multiple measured images of the same object are superimposed, noise has no significant increase, but the signal will gain after superposition. That is similar to scanning with frame averaging. Furthermore, because the background scene is not completely black during the process of imaging, specific background noise of image could be generated. Therefore, it is necessary to remove background noise. That is, if the method of superimposed measurement can be combined with the removal of imaging background noise, the image acquisition will be improved. Additionally, the mathematical model can be expressed as

(6) $$\begin{array}{ll} g(x,y) & =\eta (x,y)-n_{b}(x,y)\\ & =f(x,y)+n_{s}(x,y)-n_{b}(x,y)\\ & =f(x,y)+n(x,y), \end{array}$$

where x and y are, respectively, the number of pixels in the length and width of the image signal; g(x,y) is the measured image signal after removing the background noise; η(x,y) is the measured image signal; $n_{b}(x,y)$ is the imaging background noise; f(x,y) is the original image signal; $n_{s}(x,y)$ is the image measurement noise; and $n(x,y)=n_{s}(x,y)-n_{b}(x,y)$ is the image measurement noise after removing the background noise. Moreover, according Equation (5), the SNR of a single signal measurement with the background noise removal can be written as

(7) $$SNR_{g(x,y)}=10\log_{10}\left(\frac{\sigma_{f(x,y)}^{2}}{\sigma_{n(x,y)}^{2}}\right).$$

We measured the same image signal and the background noise several times respectively. Then, multiple sets of measured data and background noise were obtained and overlapped. The superimposed image signals can be expressed as

(8) $$\begin{array}{ll} \sum_{i=1}^{K}g_{i}(x,y) & =\sum_{i=1}^{K}\eta_{i}(x,y)-\sum_{i=1}^{K}n_{bi}(x,y)\\ & =\sum_{i=1}^{K}f_{i}(x,y)+\sum_{i=1}^{K}n_{si}(x,y)-\sum_{i=1}^{K}n_{bi}(x,y)\\ & =\sum_{i=1}^{K}f_{i}(x,y)+\sum_{i=1}^{K}\left[n_{si}(x,y)-n_{bi}(x,y)\right]\\ & =\sum_{i=1}^{K}f_{i}(x,y)+\sum_{i=1}^{K}n_{i}(x,y), \end{array}$$

where K values are measurement times, i is the number of measurement times, $g_{i}(x,y)$ is the measured data after removing the background noise of the ith measurement, $\eta_{i}(x,y)$ is the measured image signal of the ith measurement, $n_{bi}(x,y)$ is the imaging background noise of the ith measurement,$f_{i}(x,y)$ is the original image signal of the ith measurement, $n_{si}(x,y)$ is the image measurement noise of the ith measurement, and $n_{i}(x,y)=n_{si}(x,y)-n_{bi}(x,y)$ is the image measurement noise after removing the background noise of the ith measurement. The mean of the superimposed image signals can be expressed as

(9) $$\begin{array}{ll} G(x,y) & =\frac{1}{K}\sum_{i=1}^{K}g_{i}(x,y)\\ & =\frac{1}{K}\sum_{i=1}^{K}f_{i}(x,y)+\frac{1}{K}\sum_{i=1}^{K}n_{i}(x,y)\\ & =f(x,y)+\frac{1}{K}\sum_{i=1}^{K}n_{i}(x,y)\\ & =f(x,y)+N(x,y), \end{array}$$

where N(x,y) is the average noise after K times of superposition and removing the background noise. Moreover, noises in different pixels of the image are random and independent, which can be stated as

(10) $$\sum_{x=1}^{M}\sum_{y=1}^{N}\left[\frac{1}{K}\sum_{i=1}^{K}n_{i}(x,y\right)]\propto 0,$$

where the image is M×N pixels in size. According to Equation (10), the expectation of the average of the superimposed signals can be represented as

(11) E{G(x,y)}≈f(x,y).

In addition, because noises in different measurements are also independent, n(x,y) and $\sum_{i=1}^{K}n_{i}(x,y)$ follow the same distribution. Therefore, in this study we make the assumption that n(x,y) and $\sum_{i=1}^{K}n_{i}(x,y)$ tend to be the same level of intensity. Then the variance of the average of the superimposed signals can be represented as

(12) $$\sigma_{G(x,y)}^{2}=\sigma_{N(x,y)}^{2}\approx \frac{1}{K}\sigma_{n(x,y)}^{2}.$$

Substituting Equation (12) into Equation (5), the SNR of the average of the superimposed signals can be written as

(13) $$\begin{array}{ll} SNR_{G(x,y)} & =10\log_{10}\left(\frac{\sigma_{f(x,y)}^{2}}{\sigma_{N(x,y)}^{2}}\right)\\ & \approx 10\log_{10}\left(\frac{\sigma_{f(x,y)}^{2}}{\frac{1}{K}\sigma_{n(x,y)}^{2}}\right)=10\log_{10}\left(\frac{\sigma_{f(x,y)}^{2}}{\sigma_{n(x,y)}^{2}}\right)+10\log_{10}K=SNR_{g(x,y)}+10\log_{10}K. \end{array}$$

Then, subtracting Equation (7) gives us the relationship of $SNR_{G(x,y)}$ and $SNR_{g(x,y)}$, which can be written as

(14) $$SNR_{G(x,y)}\approx SNR_{g(x,y)}+10\log_{10}K.$$

**Figure 6:**
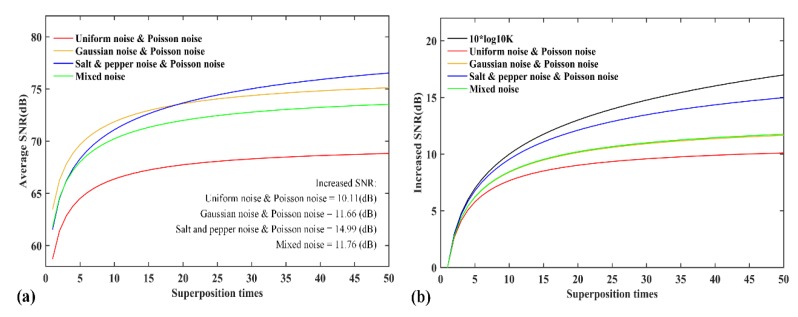
The change of SNRs for the original image signal with four different noise-dominated situations with the increase of the superposition times. (**a**) Average SNR of 50 random simulations at each number of superposition times. (**b**) The increments of SNRs in figure (**a**) with the increase of the superposition times.

To demonstrate the denoising capability of the method of superimposed measurement, we compare the changes of SNRs with different numbers of superposition times. Simulations are carried out for the same image signal with four different noises dominated, as the number of superposition times increases. A 512×512 picture with reduced grayscale is employed as the original image signal, and the measurement times K is 50. We discuss the original image signal with four different noises, which include uniform noise, Gaussian noise, salt and pepper noise, and Poisson noise. Poisson noise is used for the photon noise here. At each number of superposition times, 50 random simulations of image signal with each noise dominated were performed respectively. Figure 6 presents the change of SNRs for the original image signal with four different noise-dominated situations with the increase of the superposition times. In Figure 6, the mixed noise consists of Gaussian noise, salt and pepper noise and Poisson noise. Figure 6a shows average SNR of 50 random simulations at each number of superposition times. Figure 6b shows the increment of SNRs in Figure 6a with the increase of the superposition times. As the number of superposition times goes up, the increase of SNR becomes less and less obvious. In Figure 6b, when the number of superposition times tends to be 50, the increases of SNR values are all less than $10\log_{10}50=16.99\;(\mathrm{dB})$.

### 3.2. Image Acquisition Experiment

**Figure 7:**
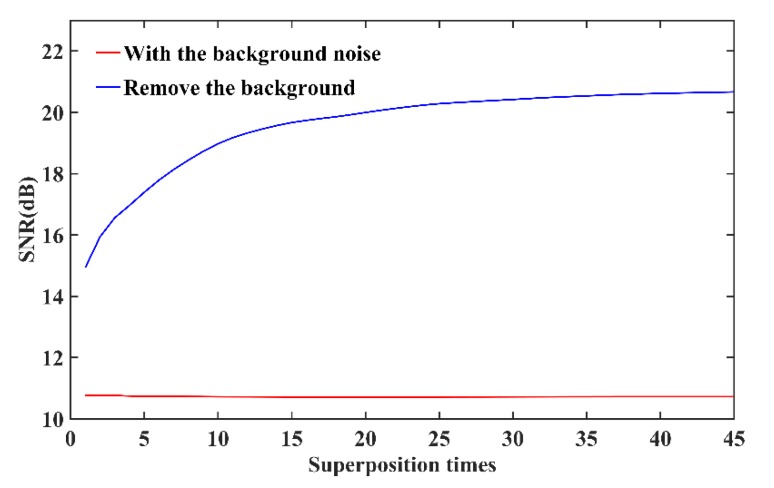
The SNR curves of the image signals with and without background noise as the number of superposition times increases.

For improving image SNR, we used the image acquisition method which was based on the statistical characteristics of image noise and the removal of imaging background noise. In our experiment, the number of superposition times was 45; that is to say, we measured the object under dim light, and 45 sets of image signals and 45 sets of background image were obtained, respectively. The reference image for calculating SNR was obtained by measuring the same object in a slightly bright light. The background image was acquired by measuring the same object with the laser dot projection system turned off. Figure 7 shows the SNR curves of the image signals with and without background noise as the number of superposition times increases. In Figure 7, with the increase of the superposition times, the SNR for the image signal removing the background noise is obviously improved, but there is no significant change in SNR for image signal with the background noise. When the image signal with the background noise removed is superimposed 45 times, the SNR can be increased by almost 5.5 dB. Compared with simulation results, the main reason for the difference in experimental results may be that the noise of each measurement is not random and independent of the others, especially for the image signal with background noise. However, for the image signal with the background noise removed, it is feasible to denoise by using the method of superimposed measurement.

## 4. Experiment

**Figure 8:**
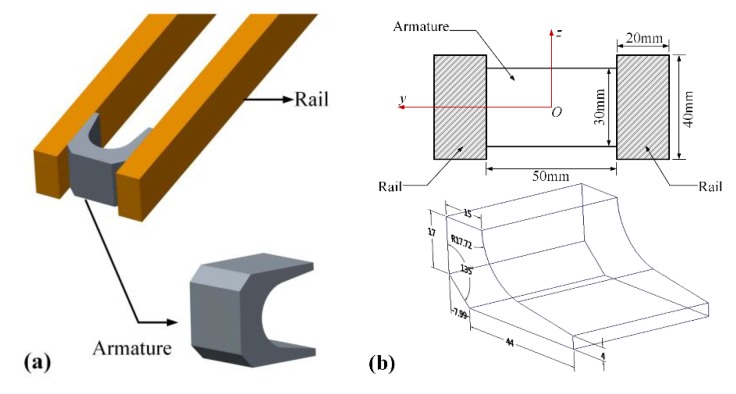
The rails and armature system. (**a**) The system structure of the rails and armature. (**b**) The sizes of the rails and the armature.

Our scanning experimental research is based on the research background of a medium caliber electromagnetic (EM) launcher, whose rails and armature system are shown in Figure 8. In Figure 8a, we show the system structure of the rails and armature, in which the armature is made from 7075 aluminum alloy, and the rails are made from copper alloy. The sizes of the rails and the armature is shown in Figure 8b. Although we focus on the rail surface after the launch, the details of the launch experiment are necessary to be known. From one launch, we know that a peak value of the discharge current profile on the breech can reach to 1000 kA, and the armature reached a muzzle velocity can be over 2000 m/s. Meanwhile, the muzzle kinetic energy was about 240 kJ.

### 4.1. Experiment System

**Figure 9:**
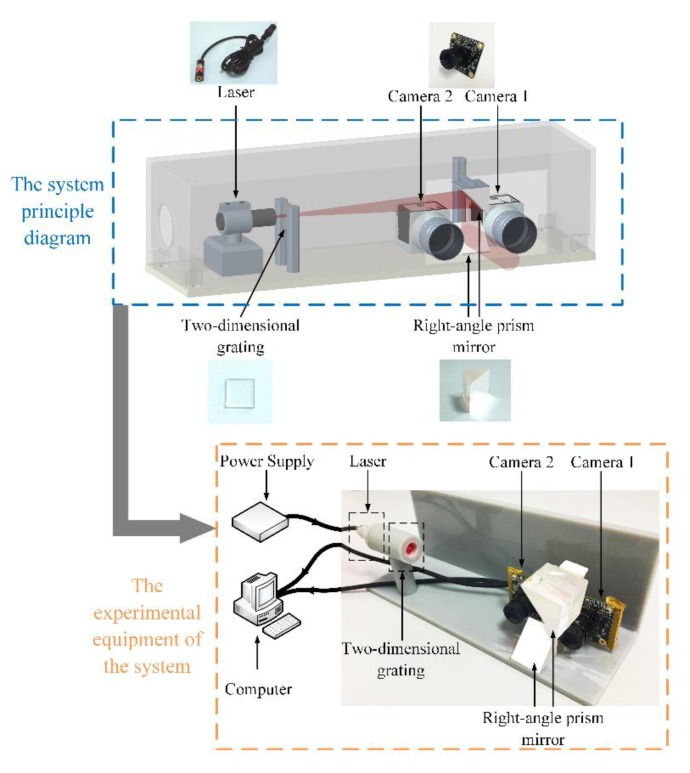
The experimental equipment of the 3D scanning system.

The experimental equipment of the 3D scanning system in Figure 9 consists of a 650 nm laser, a 15 × 15 two-dimensional grating (WLBA-694-2D-15, Shenzhen, China), two right-angle prism mirrors (Thorlabs MRA25-E02, Newton, NJ, USA), and two monochrome industrial CMOS cameras (The Imaging Source, DMK 27UP031-ML, Bremen, Germany) with 16 mm focal length. In contrast with the schematic in Figure 3, although we use two right-angle prism mirrors to better project the laser dot projection, the principle has not changed. The distance between two camera optical centers of our system is 55 mm. In addition, our system is based on the laptop computer (i5-6700K) and Matlab for image processing and 3D surface reconstruction.

### 4.2. Stereo Calibration

**Figure 10:**
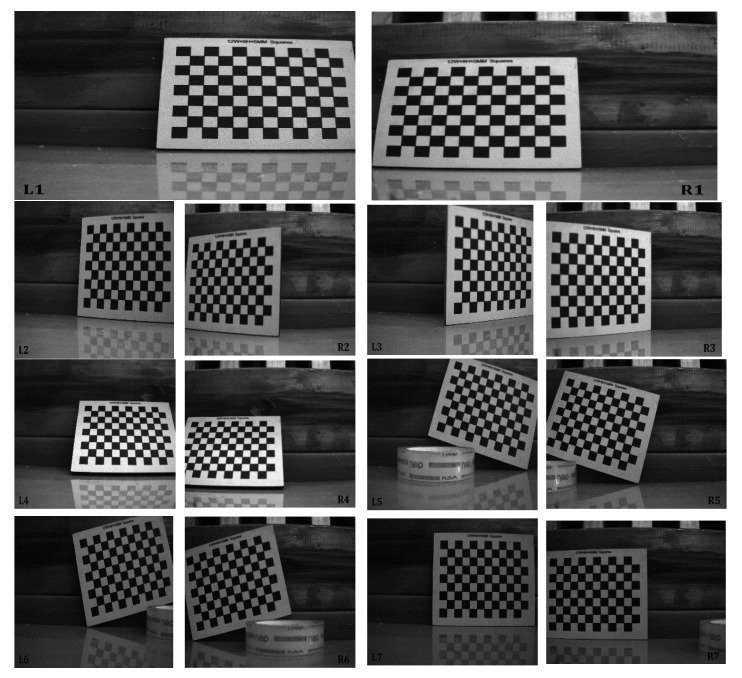
Seven sets of images of calibration boards of different poses obtained by the left and right cameras. Figures, **L1**–**L7** were captured by left camera. Figures, **R1**–**R7** were captured by right camera.

The proposed 3D scanning system is based on binocular stereo vision. Therefore, in order to ensure the matching accuracy of laser dots and obtain more accurate 3D reconstruction results, binocular stereo calibration is the necessary thing. Here, we used the stereo calibration method proposed by Z. Zhang [18,19] combined with calibration board to obtain cameras’ internal parameters, cameras’ distortion coefficients, and the relative position relationship between the left and right cameras. Additionally, for calibration, the left and right cameras collected seven sets of images. In each set of images, the calibration board was under different postures. The calibration images are shown in Figure 10.

Based on Zhang’s camera calibration, with the seven sets of calibration images, we got the internal parameters shown in Table 1 and external parameters shown in Table 2 of both two cameras, such as the focal length, distortion coefficients of the camera, the rotation vector, and the translation vector.

In Table 1, $a_{x}$ and $a_{y}$ denote the camera focal length, respectively, in X direction and Y direction; $x_{0}$ and $y_{0}$ denote the principal point, respectively, in X direction and Y direction; ac is the nonvertical factor; and $k_{1}$, $k_{2}$, $k_{3}$, $k_{4}$, and $k_{5}$ are the distortion coefficients. In Table 2, $R_{0}$ denotes the rotation vector, and T denotes the translation vector.

According to the internal parameters and external parameters of binocular camera, left and right images obtained by the binocular camera can be calibrated; that is, the errors caused by camera distortion, focal length error, and binocular camera position can be corrected, greatly improving the accuracy of 3D reconstruction.

Firstly, the distortion of left and right images can be calibrated by the internal parameters of binocular camera. The coordinates of acquired images are remapped to calibrate the distortion. If the original coordinate is represented as $r^{2}=x^{2}+y^{2}$, the calibrated image coordinates $\left(x^{\prime },y^{\prime }\right)$ can be written as [19,20,21].

(15) $$\left[\begin{array}{c} x^{\prime }\\ y^{\prime } \end{array}\right]=\left(1+k_{1}r^{2}+k_{2}r^{4}+k_{5}r^{6}\right)\left[\begin{array}{c} x\\ y \end{array}\right]+dx\;,$$

where dx is the tangential distortion vector, and it can be expressed as

(16) $$dx=\left[\begin{array}{c} 2k_{3}xy+k_{4}\left(r^{2}+2x^{2}\right)\\ k_{3}\left(r^{2}+2y^{2}\right)+2k_{4}xy \end{array}\right]\;.$$

The five distortion coefficients, $k_{1}$, $k_{2}$, $k_{3}$, $k_{4}$, and $k_{5}$ are used to calibrate the radial and tangential distortion of each camera. After the distortion calibration is completed, the coordinates of images acquired by cameras need to be re-projected, which will be carried out by the camera matrix, and the process can be expressed as

(17) $$\left[\begin{array}{c} x^{new}\\ y^{new}\\ 1 \end{array}\right]=K\left[\begin{array}{c} x^{\prime }\\ y^{\prime }\\ 1 \end{array}\right]\;,$$

(18) $$K=\left[\begin{array}{ccc} a_{x} & \mathrm{ac}\times a_{x} & x_{0}\\ 0 & a_{y} & y_{0}\\ 0 & 0 & 1 \end{array}\right]\;,$$

where K is the camera matrix.

Furthermore, the external parameters of the binocular camera are also calibrated by a rotation vector and a translation vector. The rotation vector can be converted into a rotation matrix by Rodrigues’ rotation formula. If $P\left(x_{w},y_{w},z_{w}\right)$ denotes a point in the world coordinate system, the mapping coordinates $\left(x_{w},y_{w},z_{w}\right)$ of the left and right cameras will satisfy the following equation:

(19) $$\left[\begin{array}{c} x_{r}\\ y_{r}\\ z_{r} \end{array}\right]=\left[\begin{array}{cc} R & T\\ 0^{T} & 1 \end{array}\right]\left[\begin{array}{c} x_{l}\\ y_{l}\\ z_{l} \end{array}\right]\;,$$

where R is the rotation matrix transformed from the rotation vector, T is the translation vector, $\left(x_{l},y_{l},z_{l}\right)$ are the mapping coordinates $\left(x_{w},y_{w},z_{w}\right)$ of the left camera, and $\left(x_{r},y_{r},z_{r}\right)$ are the mapping coordinates of the right camera. The rotation matrixes of left and right cameras satisfy the following equation:

(20) $$R=R_{l}\times R_{r},$$

where $R_{l}$ is the rotation matrix of left camera and $R_{r}$ is the rotation matrix of right camera.

**Figure 11:**
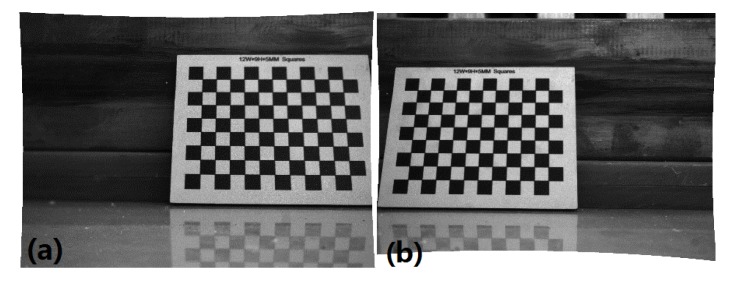
Images after calibration. (**a**) Figure 10L1 after calibration. (**b**) Figure 10R1 after calibration.

According to the internal and external parameters of the binocular camera, we calibrated a set of images, which were the first set of calibration images (Figure 10L1 and R1) in Figure 10. Additionally, images after calibration are shown in Figure 11.

### 4.3. Result and Discussion

**Figure 12:**
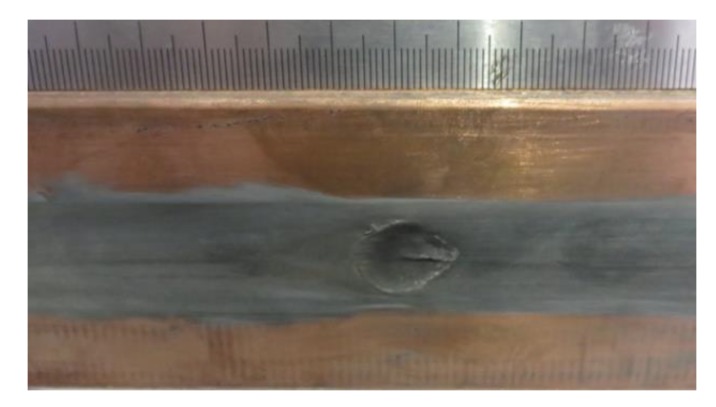
A part of the rail surface containing a gouge crater.

In order to verify the feasibility of the 3D surface scanning system, a group of surface scanning tests were conducted after several railgun launch experiments. A gouge crater on the rail surface shown in Figure 12 was scanned out under dim light. The gouge crater is a representative surface damage of the rails after the launch.

**Figure 13:**
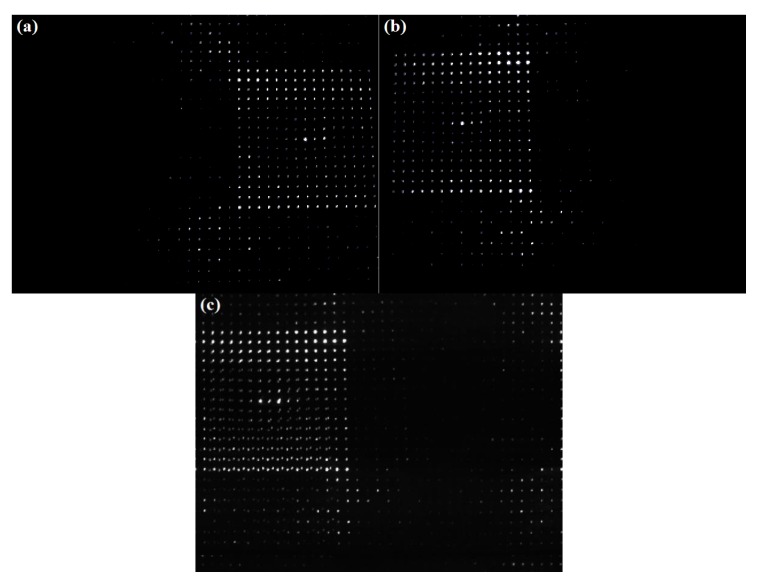
Surface images containing laser dots in dim light condition. (**a**) Processed image captured from Camera 1. (**b**) Processed image captured from Camera 2. (**c**) The matching results of figure (**a**) with respect to figure (**b**).

When we started scanning, Camera 1 and Camera 2 captured two surface images in dim light respectively. Additionally, for high image SNR, we used the image acquisition method which we proposed. We sampled the same position of the rail surface using the same laser dots pattern multiple times to improve the SNR. As known from the high SNR image acquisition experiment, when the sampling frequency is greater than 20, the SNR improvement tends to be stable. Thus, we measured the rail surface under dim light 20 times, and 20 sets of image signal from Camera 1, 20 sets of background images from Camera 1, 20 sets of image signals from Camera 2, and 20 sets of background images from Camera 2 were obtained, respectively. Then each set of image signal from Camera 1 and Camera 2 subtracted its corresponding background image. Additionally, by overlapping 20 times and averaging, we acquired two images, which are shown in Figure 13a,b respectively. Meanwhile, a regional fuzzy binocular stereo matching algorithm based on global correlation coding was used for the two images’ laser dot matching [22]. Additionally, Figure 13c shows the matching results of Figure 13a with respect to Figure 13b. In Figure 13, we can see not only the 15 × 15 dots, but also other weaker dots, because of the diffraction effect of the grating. Accordingly, the computer image processing technology was utilized to carry on binary processing for the two images, which thereby made the 15 × 15 dots clearer. After that, the coordinates of centroid pixels were extracted by using the centroid algorithm. Because the centroids of the dots in the two processed images were feature points, according to Equation (2), the distances of the feature points were acquired.

**Figure 14:**
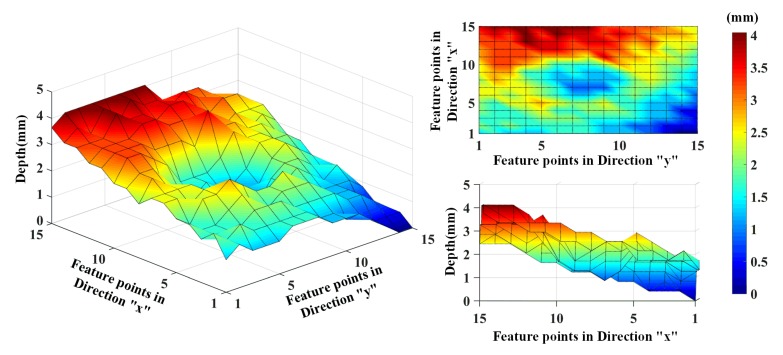
The 3D depth map of the rail surface with the depth information of the 15 × 15 feature points.

We select the point which has the maximum distance, and define that the plane of that maximum point is the reference plane. Then the distances between the reference plane and these feature points are calculated. Therefore, we describe a depth map of the rail surface relative to the reference plane. Figure 14 shows the 3D depth map of the rail surface with the depth information of the 15 × 15 feature points. We can see a reconstructed gouge crater in the depth map, and the gouge crater is about 1.5 mm in depth. Meanwhile, viewed from the side view of the reconstructed surface, the reconstructed results are slightly slanted; this is because the system was not calibrated.

**Figure 15:**
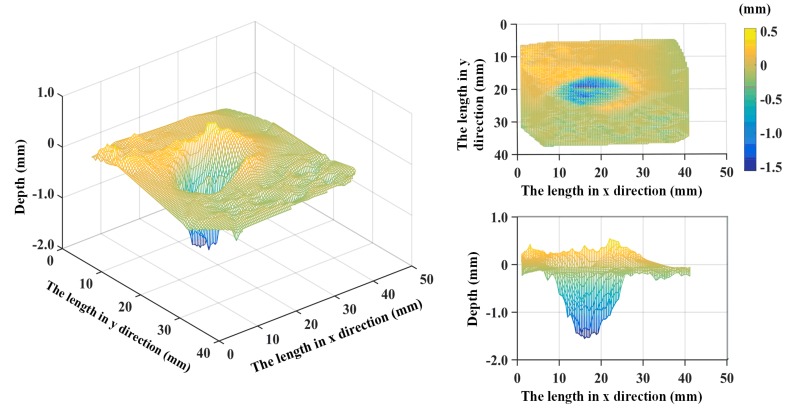
The 3D depth map of the rail surface with the depth information of several experiments.

After calibrating the system, we carried out many scanning experiments on this gouge crater. In each experiment, the number of the dot matrix was the same, but the position of the dot matrix of each scan was a little different from the previous one, with a little offset. Additionally, in each experiment, the laser dots pattern could cover the whole gouge crater. We chose one of the experiments as the reference and took the plane of the point with the smallest distance as the reference plane. Then, based on the binocular vision calibration, the depth map of the rail surface relative to the reference plane can be described. Figure 15 shows the 3D depth map of the rail surface with the depth information of several experiments. We can see a reconstructed gouge crater in the depth map, and the gouge crater is about 1.5 mm in depth.

As mentioned above, the proposed 3D scanning system is feasible as the means to measure the complex rail surface. Additionally, the experiment was done in weak light, just like the in-bore environment.

### 4.4. System Evaluation and Measurement Uncertainty Analysis

#### 4.4.1. System Evaluation

In 3D measuring system, the measurement resolution, as an indicator of system evaluation, concerns the design and implementation of the whole system. Thomas Luhmann was provided a simplified analysis method for the binocular stereo vision system [23]. This method was applied to analyze the influence of variation of parallax on location of object coordinates. This method assumed that the baseline length and the focal length had no measurement errors. Then the variation estimation of the object depth information can be given by differentiation of Equation (2)

(21) $$s_{z}=\frac{z^{2}}{T\cdot f}s_{d}=\frac{z}{T}\cdot \frac{z}{f}s_{d}$$

where $s_{z}$ is the variation in the viewing direction and $s_{d}$ is the variation of parallax measurement. Equation (21) is the partial of *z* with respect to *d* in Equation (2). Equation (21) also shows that the variation in the viewing direction is a function of the variation of parallax measurement. When the parallax value is a unit pixel, the depth information measured by the system will be the minimum depth change which can be measured; that is, the resolution of the scanning system.

In our works, since our experiment is just a proof-of-principle experiment, the maximum distance between cameras and the rail surface is about 150 mm. The base length is 55 mm, and the focal length is 16 mm. In addition, from the basic parameters of the camera, we know that the single pixel width is 2.2 μm. That is, if the base length and the focal length are assumed free of error, according to Equation (21), the measurement resolution of the system is approximately 56 μm.

#### 4.4.2. Measurement Uncertainty Analysis

**Figure 16:**
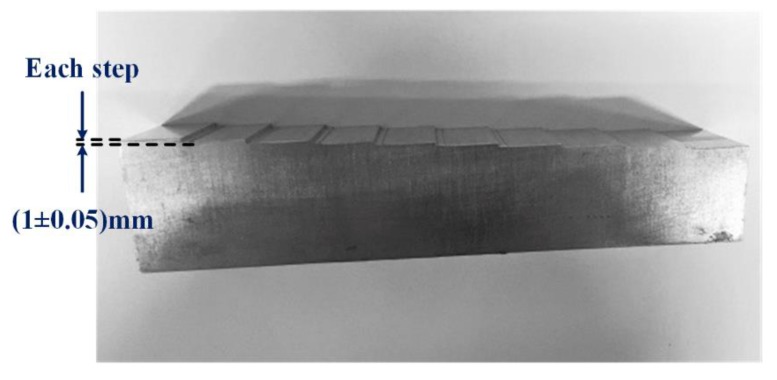
The high precision standard parts used for measurement accuracy evaluation.

To further evaluate the measurement data of the 3D scanning system, we used a high precision standard parts shown in Figure 16. The height of each step of the standard parts was 1 mm, and the machining accuracy of each step at the design time was 1 ± 0.05 mm. Then the 3D scanning system was used to scan and measure the steps of the standard parts eight times. The measurement results and the measurement value of each step are shown in Table 3. As we can see in Table 3, the average of each step’s value is 0.9976 mm.

In order to further evaluate the measurement results, uncertainty of measurement is used. According to the guide to the expression of uncertainty in measurement (GUM), combined with Table 3, type A standard uncertainty can be expressed as

(22) $$u_{A}=\sqrt{\frac{\sum_{i=1}^{7}{\left(x_{i}-\bar{x}\right)}^{2}}{(7-1)\times 7}}=0.012\mathrm{mm}$$

Then, according to the machining accuracy of each step of the standard parts, we know that ±0.05 mm defines an interval having a level of confidence of 95 percent. Therefore, type B standard uncertainty can be expressed as

(23) $$u_{B}=\frac{0.05}{k_{95}}=\frac{0.05}{1.96}=0.026\mathrm{mm}$$

where $k_{95}$ is the factor corresponding to a 95-percent level of confidence. Additionally, from GUM, we know that the factor corresponding to a 95 percent level of confidence is 1.96. According to Equations (22) and (23), type A standard uncertainty and type B standard uncertainty are obtained respectively. Therefore, the combined standard uncertainty is

(24) $$u_{C}=\sqrt{u_{A}{}^{2}+u_{B}{}^{2}}=\sqrt{0.012^{2}+0.026^{2}}=0.03\mathrm{mm}$$

Additionally, the measurement result of each step of the standard parts with the combined standard uncertainty is 0.998 ± 0.03 mm, which is close to the true value.

## 5. Conclusions

In summary, to detect the rail surface profile of the railgun under complex inner bore environment, we have demonstrated a feasible system based on the binocular stereovision theory and laser dot projection. Compared with the 3D measurement methods which are based on fringe projection active illumination, our system is less affected by the complex inner bore environment and can reconstruct the object more completely. Moreover, an image acquisition method is used to improve the image SNR, which is based on the statistical characteristics of image noise and the removal of imaging background noise. The results of the superposition experiment suggest that the image SNR can be increased by almost 5.5 dB or even more. We have done the proof-of-principle experiment for the system on a disassembled rail under specified conditions. In the experiment, a part of the rail surface containing a gouge crater has been scanned under dim light. The system adopts the binocular vision technology combined with laser dot projection, in which laser dot projection is used as active illumination. Meanwhile, the centroid algorithm is applied to extract the center pixel of laser dots, which are used as feature points for binocular matching. As can be seen from the test results, we describe the 3D depth map of the rail surface and show that the gouge crater is about 1.5 mm in depth. Meanwhile, we have made some theoretical analyses on the measurement resolution of the system, and results showed that the resolution of the system is about 56 μm. Furthermore, we analyzed the measurement uncertainty of a set of measurement results, and the analysis showed that the measurement result was close to the true value. Therefore, it is proven to be feasible, through experiments, that our 3D scanning system can measure the complex rail surface in weak light. Although this experiment was still an external experiment by moving the rails out, we fully simulated the complex inner bore environment of the railgun. Additionally, our expectation is that such a 3D scanning system can be used in the bore of electromagnetic railgun without disassembling the rails. In our future work, the system needs to be improved. We will be committed to the algorithm of the system refinement and the improvement of system in engineering applications, such as a 3D scan of the rail surface in the bore of railgun without disassembling the rails, and automatic detection of flaws starting from acquired 3D data.

## Figures and Tables

**Table 1:** Internal parameters and errors of binocular camera.

Internal Parameters	Left Camera	Right Camera
*α*_x_	3807.64 ± 26	3774.53 ± 22
*α*_y_	3806.41 ± 25	3768.74 ± 20
*x*_0_	1360.88 ± 27	1266.53 ± 26
*y*_0_	897.44 ± 23	931.05 ± 21
*ac*	0.00 ± 0.00	0.00 ± 0.00
*k*_1_	−0.40 ± 0.037	−0.31 ± 0.032
*k*_2_	0.45 ± 0.19	−0.22 ± 0.14
*k*_3_	0.0080 ± 0.0015	0.0020 ± 0.0012
*k*_4_	−0.0037 ± 0.0030	0.0023 ± 0.0028
*k*_5_	0.00 ± 0.00	0.00 ± 0.00

**Table 2:** External parameters of binocular camera.

External Parameters	X Direction	Y Direction	Z Direction
**Rotation vector** $R_{0}$	−0.03963	−0.03602	−0.01476
**Translation vector** T	−39.15007	0.63651	−0.16640

**Table 3:** Measurement results of the standard parts.

Number of Measurement Times	Measurement Value (mm)	Number of the Step *i*	Each STEP’ s Value *x_i_* (mm)	Average of Each Step’s Value $\bar{x}$ **(mm)**	Residual $x_{i}-\bar{x}$ **(mm)**
1	1.1398			0.9976	
2	2.1749	1	1.0351		0.0375
3	3.1308	2	0.9559		−0.0417
4	4.1411	3	1.0103		0.0127
5	5.1062	4	0.9651		−0.0325
6	6.1426	5	1.0364		0.0388
7	7.1221	6	0.9795		−0.0181
8	8.1232	7	1.0011		0.0035
